# Pain nursing for gynecologic cancer patients

**DOI:** 10.3389/fonc.2023.1205553

**Published:** 2023-07-26

**Authors:** Wei Wu, Xiaodan He, Shenjie Li, Ming Jin, Yali Ni

**Affiliations:** Department of Gynecology, Cancer Hospital of China Medical University, Liaoning Cancer Hospital and Institute, Shenyang, Liaoning, China

**Keywords:** gynecology, malignant tumor, cancer pain, nursing, psychology of pain

## Abstract

Gynecological malignancy remains a prevalent cause of mortality among women. Chronic cancer pain, as a severe complication of malignancy and its therapies, accounts for a substantial burden of physical and psychological distress in affected patients. Accordingly, early identification, assessment, and standardized management of such pain are crucial in the prevention or delay of its progression. In the present review, we provide a comprehensive overview of the pathological factors that contribute to pain in patients with gynecological malignancy while highlighting the underlying mechanisms of pain in this population. In addition, we summarize several treatment modalities targeting pain management in gynecologic cancer patients, including surgery, radiotherapy, and chemotherapy. These interventions are crucial for tumor elimination and patient survival. Chronic cancer pain exerts a significant impact on wellbeing and quality of life for patients with gynecologic cancer. Therefore, our review emphasizes the importance of addressing this pain and its psychological sequelae and advocates for a multidisciplinary approach that encompasses nursing and psychological support. In summary, this review offers valuable insights into the pathological factors underlying pain, reviews pain management modalities, and stresses the critical role of early intervention and comprehensive care in enhancing the quality of life of these patients.

## 1 Introduction

Gynecological malignancies are among the most prevalent types of female malignancies, with high incidence rates in the ovarian, cervix uteri, and uterine bodies (1). Among these, cervical cancer has the highest incidence rate, followed by endometrial and ovarian cancers. These malignancies are known to cause significant morbidity and mortality in females, with lung cancer being the only type of malignancy with higher incidence and mortality rates (2, 3). Treatments such as surgery, radiotherapy, and chemotherapy have been known to improve the survival time of gynecological malignancy patients by removing the tumor and regional lymph nodes. However, these treatments also lead to severe impacts on the patient’s quality of life (4–7). Cancer pain is among the critical factors affecting the patient’s quality of life. Around 70% of patients suffering from advanced gynecological malignant tumors reported experiencing varying degrees of cancerous pain (8). The pain is caused by the tumor, metastases, cancer embolus, and infiltration into the surrounding tissues (9). Cancer pain is universal and severe, and is often difficult to manage, leading to the need for continuous drug intervention. The incidence rate of cancer pain is 50.7% in all cancer stages and 66.4% in advanced stages (10).

Cancer-related pain is a highly prevalent subjective symptom observed in patients with gynecologic malignancies (11). The nature of pain experienced by patients is generally dull and of a moderate degree. The pain predominantly affects the pelvic cavity and adjacent tissues, including the hypogastrium, perineum, and waist. Additionally, several patients experience pain in more than two sites. Cancer-related pain is a burdensome issue for patients and may lead to various adverse outcomes, such as interference with daily activities, hindrance of social function, and impairment of cognitive function (12). The consequences of cancer-related pain can negatively impact patients’ quality of life, leading to pessimism, anxiety, fear, and depression, resulting in patients’ societal and economic dependence. Furthermore, pain may affect patients’ sleep quality, leading to adverse emotions and reduced disease resistance, which is potentially detrimental to tumor treatment and symptom relief. Therefore, healthcare professionals are advised to pay adequate attention to the management of cancer-related pain in patients with gynecologic malignancies, including an accurate and reasonable evaluation and intervention. In this review, we focused on pain nursing for gynecologic cancer patients, emphasizing the importance of early identification, evaluation, and standardized pain treatment. We summarized the pathological factors contributing to pain in this population and reviews the pain management regimen for gynecologic cancer patients. Regarding the search strategies, we utilized a systematic approach to identify relevant studies, including searching electronic databases (Pubmed, Cochrane Library and Web of science), reviewing reference lists, and consulting with experts in the field. We also established inclusion and exclusion criteria to select studies for review and used methods to analyze and synthesize the data.

## 2 Pathophysiology of chronic pain in gynecological cancer

Gynecological malignancies encompass tumors that occur in various regions such as the cervix, endometrium, ovary, and other areas (**Table 1**). Among women, cervical cancer, endometrial cancer, and ovarian cancer are the most prevalent gynecological malignancies (13, 14). These malignancies are characterized by symptoms such as increased leucorrhea with an unpleasant odor, vaginal bleeding, the lump of hypogastrium, bearing-down pain, low fever, weight loss, and other indications, which can impede the health and well-being of patients (4). The pathogenesis of gynecological tumors remains widely unknown, and in most cases, these malignancies do not manifest apparent symptoms during the early stage. Distinguishing between benign and malignant malignancies preoperatively is difficult, and detection typically occurs during the moderate to late stages of the disease, significantly impacting the patient’s treatment and survival (2, 15). In recent years, various new concepts, new methods and new technologies have continuously emerged medicine (16, 17). Advancements in medicine continue to introduce novel concepts, methods, and technologies. Researchers believe that several factors, including reproductive history, menstrual history, sexual behavior, sexual age, endocrine function anomalies, lifestyle choices such as smoking, drinking, and infections, can increase the likelihood of developing gynecological malignancies (18, 19). Studies have shown that an unhealthy lifestyle, abnormal endocrine function, and sexual behaviors are high-risk factors for gynecological malignant tumors, which have close correlation with the incidence of cancer (20). The main therapeutic approaches and diagnostic markers are listed in **Tables 2** and **3**.

**Table 1:** The features of gynecologic cancer.

Cancer type	Symptoms		Diagnostic methods	Susceptible factors
	Early stage	Advanced stage		
Cervical cancer	menostaxis, menorrhagia, colporrhagia and vaginal drainage	anemia, cachexia, multiple organ failure	cervical cytology	early sexual age and casual sex
				multiple pregnancies and deliveries
Endometrial cancer	irregular vaginal bleeding, vaginal drainage,	abdominal mass, paroxysmal hypogastric pain	cervical cytology	Abnormal hormone secretion
				Bad living habits
Ovarian cancer	persistent distending pain, irregular menstruation	emaciation, lump in the lower abdomen, ascites	cervical cytology	Carcinogenic factor
				Bad living habits and genetic factors

**Table 2:** The comprehensive diagnosis of gynecologic cancer.

Cancer type	Early screening	Locating diagnosis	The qualitative diagnosis	Clinical staging
Cervical cancer	Cervical/vaginal abscission cytology and HPV (human papillavirus) DNA testing and colposcopy	Ultrasonography, computed tomography (CT), magnetic resonance imaging (MRI), uterine coegg tube angiography (HSG), arteriography and lymphography.	Embryonic and placental tumor markers	lymphography
			Glycochain antigen tumor markers	
			Enzyme and isozyme tumor markers	
Endometrial cancer	Endometrial cytological screening, hysteroscopic biopsy, and pathological confirmation; Color Doppler ultrasound	Color Doppler flow imaging, Transvaginal ultrasonography,	Hormone bean hormone receptor tumor marker	Radiological interventional technique
			Tumor related material markers	Laparoscopic operation
			cytokines	
Ovarian cancer	Doppler ultrasound	Interventional ultrasound	Virus marker	
			Gene tumor marker	

**Table 3:** The chemotherapy of gynecologic cancer.

Cancer type	Main chemotherapeutics	Indications
Cervical cancer	BIP (cisplatin + bleomycin + isophoramide),	Locally advanced cervical cancer I b-II a period
	PT (carboplatin/cisplatin + paclitaxel),	with high risk factors of early cervical cancer
	VBP (vincristine + cisplatin + bleomycin)	(such as local tumor diameter 4 cm or higher)
	Concurrent chemoradiotherapy	parauterine invasion, lymphatic vascular space involvement,
	Platinum-based chemotherapy	positive surgical margin, local progression,
		extensive lymph node and systemic metastasis and recurrence
	Postoperative adjuvant chemotherapy	cervical cancer I a-II a period
Endometrial cancer	Carboplatin and paclitaxel	Early high-risk (G3 grade) tumors, myometrial
		invasion of > 1/2, and/or cervical interstitial involvement)
Ovarian cancer	Taxol + carboplatin (TC regimen)	Serous and mucinous carcinomas
	Cisplatin/carboplatin + paclitaxel	The diameter of peritoneal metastases was 5-20 cm

### 2.1 Cervical cancer and pathogenic factors

Cervical cancer is the most frequently occurring malignancy in the female genital tract (21, 22). During the early stages of the disease, no definite symptoms or signs are apparent. However, as the disease advances, various symptoms arise, such as menostaxis, menorrhagia, colporrhagia, and vaginal discharge. The extent of infiltration of the cancerous cells causes the emergence of secondary symptoms, and frequently observed symptoms in the advanced stages comprise cachexia, anemia, and multiple organ failure (23–25). Several factors influence the incidence of cervical cancer, including smoking, infection by high-risk strains of human papillomavirus (HPV), multiple sexual partners, an early onset of menstruation, a history of multiple pregnancies and deliveries, together with infections from pathogenic agents such as Chlamydia trachomatis, Herpes simplex, Type II Trichomonas vaginalis, and trichomonads. Cervical cancers in more than 90% of cases are associated with high-risk HPV infections (26, 27). A significantly higher incidence of cervical cancer was evident among females infected with strains such as 26, 53, 66 than uninfected individuals. Recent research findings indicate that commencing sexual activities at an early age, particularly under the age of twenty, can substantially heighten the risk of cervical cancer (28–30). During pregnancy, an increase in estrogen and progestational hormone levels is liable to trigger various adverse effects on the cervical environment, thus escalating the danger of cancer (31).

### 2.2 Endometrial cancer and pathogenic factors

Endometrial cancer is a malignant tumor that arises from the epithelial lining of the endometrium, which is the inner layer of the uterus. It ranks second among female reproductive malignancies and is the third most common cause of death in women (32, 33). The disease is frequently asymptomatic in the early stages, and incidental discovery during general or gynecological examination is common. As the disease progresses, patient may experience various symptoms, including irregular vaginal bleeding, vaginal discharge, abdominal mass, and paroxysmal hypogastric pain, which pose a significant risk to the patient’s health (34, 35). Endometrial cancer incidence is higher among perimenopausal and postmenopausal women. While the disease is primarily related to hormonal factors, menstrual and reproductive history, and lifestyle factors are also involved (35, 36). Dysregulation of the uterine endocrine system, such as progesterone deficiency and estrogen excess, can lead to continuous stimulation of the endometrium, resulting in endometrial hyperplasia, menstrual irregularities, and eventually, endometrial cancer (37). Epidemiological studies have demonstrated that unhealthy lifestyle behaviors such as overeating, smoking, and alcohol consumption are significantly associated with endometrial cancer. Patients with an unhealthy lifestyle exhibit lower estrone and estradiol levels while having a higher incidence of endometrial cancer (38–40). Moreover, recent studies suggest that certain reproductive factors, such as delayed menopause, nulliparity, and never have given birth, are related to endometrial cancer development. Delayed menopause prolongs the endometrial exposure to estrogen, consequently increasing the risk of developing the disease. Conversely, pregnancy protects the endometrium from estrogen stimulation and consequently reduces endometrial cancer risk (41, 42).

### 2.3 Ovarian cancer and pathogenic factors

Ovarian cancer is the third most common malignancy affecting the female reproductive system, with the highest mortality rate among all types of gynecological tumors (43, 44). The clinical manifestations of ovarian cancer include persistent distending pain, irregular menstruation, emaciation, a lump in the lower abdomen, and ascites (45). This type of cancer mainly occurs in perimenopausal women, and its etiology remains obscure due to the complex interactions between embryonic development and endocrine function. It is generally attributed to dietary and nutritional disorders, poor living habits, chemical, physical, and carcinogenic biological factors, endocrine, hereditary, and psychological factors (46, 47). Research studies have indicated that women who experience early menstruation and delayed menopause are more susceptible to ruptures on the ovarian surface during each ovulation. Abnormal repairs that occur during the subsequent repair process, owing to the decline in endocrine function, can increase the risk of ovarian cancer (48, 49). Furthermore, studies have demonstrated an association between ovarian cancer and adverse emotional states, such as anxiety and depression. These emotions may adversely affect the body’s endocrine system, leading to an imbalance in ovarian endocrine function and ultimately resulting in cancer (50, 51). In addition, research has shown that the use of hormone drugs is also associated with a higher incidence of ovarian cancer (52, 53).

## 3 Assessment and diagnosis of pain

Patients experiencing cancer pain often report it as a subjective feeling that is evaluated comprehensively based on its location, degree, and duration. Various assessment tools are commonly used in the clinic, such as McMillan’s Pain Assessment Form, Visual Analog Scale, and Wong-Banker Facial Expression Scale. The selection of an appropriate assessment method should depend on the patient’s physical condition, cultural background, and level of consciousness (54).

### 3.1 The mechanism of chronification process of pain

The chronification process of pain in gynecological cancer patients is complex and involves the interaction of various mechanisms. Firstly, ongoing tissue damage and inflammation can lead to the sensitization of peripheral nociceptors, resulting in increased pain sensitivity and decreased pain thresholds. This can lead to a self-perpetuating cycle of pain, as the continuous stimulation of nociceptors can lead to changes in their excitability and trigger the release of pro-inflammatory mediators, intensifying the pain response (55, 56). Secondly, the central nervous system (CNS) can undergo neuroplastic changes in response to chronic pain. These changes can lead to altered pain perception, making pain feel more intense and difficult to control. Neuroplastic changes can occur at various levels of the CNS, including the spinal cord and brain, and can involve altered activity in pain-processing regions and disrupted inhibitory pathways (57, 58). Thirdly, psychosocial factors such as anxiety, depression, and stress can also contribute to the chronification of pain in gynecological cancer patients. These factors can affect pain perception and increase pain sensitivity, as well as impair coping strategies and quality of life. The relationship between pain and psychosocial factors is bidirectional, with pain exacerbating psychosocial symptoms, and psychosocial symptoms exacerbating pain (59, 60). These mechanisms work together to contribute to the chronification of pain in gynecological cancer patients. Effective pain management strategies in these patients may involve a multifaceted approach that targets peripheral and central sensitization, as well as psychosocial factors (61).

### 3.2 Standardized management of pain

Pain is a pervasive phenomenon that can cause physical discomfort and psychological distress in patients, ultimately leading to a reduction in their overall quality of life. Effective pain control is, therefore, of paramount importance for ensuring the well-being of patients. Nurses play a crucial role in pain management and their care has been shown to yield significant benefits (62). In recent years, many hospitals have been implementing pain management measures to minimize negative outcomes in physical and psychological states. Pain management aims to optimize analgesic efficacy while minimizing adverse drug reactions and avoiding inappropriate drug administration (63, 64). There have been two significant shifts in the management of pain in cancer patients. Firstly, there was a transition from pain control to pain management. Secondly, there has been a gradual shift in the personnel involved in pain management, from the original anesthesiologist-based treatment model to the nurse-based care model (65). Nurses should be responsible for pain assessment, analgesic treatment, education, and promotion. Presently, cancer pain management faces many challenges due to the multifactorial and complex nature of cancer pain itself. As a result, clinical management of this particular type of pain primarily relies on drugs (66, 67). Acceptable treatment options for cancer pain in patients include over-the-counter analgesics, non-opioid prescription medications, interventions, complementary therapies, and systemic opioids. Although the World Health Organization (WHO) recommends the three-step analgesic method due to its effectiveness, some patients may seek non-pharmacological treatment due to an aversion to treatment-related adverse effects and a fear of developing addiction (68). Studies have explored the roles of various integrative medicine therapies, such as mind-body practices, acupuncture, massage therapy, and music therapy, in cancer pain management. Data from randomized controlled trials have provided support for the efficacy of hypnosis, acupuncture, and music therapy in reducing pain (69). Nonetheless, 60% of patients still suffer from pain that cannot be effectively managed due to the diverse symptoms they experience despite the development and study of multiple pain management strategies and approaches (70).

### 3.3 Standardized diagnosis and treatment system in pain clinic

Effective pain management is a critical aspect of cancer patients’ rehabilitation and palliative care. The pain clinic’s management plays an essential role in ensuring standardized pain diagnosis and treatment. It functions as a crucial link and bridge between inpatient and outpatient care. Standardized clinic treatment can enhance the efficacy of treatment while improving patient satisfaction with the quality of medical care (70). The development of pain clinics can facilitate pain control, thereby enhancing the quality of pain diagnosis and treatment services provided to outpatients. Therefore, outpatient management is of utmost importance in the standardized treatment of pain.

## 4 Management of pain

### 4.1 Integrative care of cancer pain treated with drugs

As per the treatment protocol for cancer pain recommended by the World Health Organization (WHO), non-opioid analgesics, weak opioids, and strong opioids are prescribed for patients with mild, moderate, and severe advanced cancer pain, respectively. The representative drugs for each category are aspirin, codeine, and morphine, respectively (71, 72). During medication, it is imperative to adhere to the prescribed dosage and follow the doctor’s instructions. Patient-specific pain evaluation is crucial to customize the treatment plan, and the selection of analgesic drugs should follow the principle of gradual escalation from weak to strong and from fewer to more potent medications. Whenever feasible, oral medications should be considered to minimize the risk of intravenous complications, ensure improved pain management, and adherence to medication (73). The treatment outcomes and adverse effects should be vigilantly documented, and the management of any associated symptoms should be addressed accordingly. If necessary, medication changes may be considered.

### 4.2 Integrative care for non-drug treatment of cancer pain

Non-pharmacological interventions are recommended for management of cancer pain, which include minimally invasive interventional therapy and physical therapy. Pain due to tumor infiltration and invasion of peripheral sensory nerves is characterized by acuteness, persistence, and progressive aggravation. Mild stimulation can cause severe pain, which can seriously affect patients’ psychological and physiological functions and reduce patient compliance with treatment, posing a barrier to comprehensive treatment of patients (74, 75). For such patients, minimally invasive interventional therapy is recommended. Various physical or chemical methods can be utilized to block the corresponding sensory nerves, under guidance of ultrasound, C-arm X-ray machine or computer tomography, to effectively eliminate malignant pain and manage clinical symptoms (76, 77). Nursing staff should actively collaborate with clinical doctors to provide adequate treatment and care for patients. Accurate judgment of the nature and location of the cancer pain is important to carry out preoperative preparation, implement thorough disinfection protocols, carefully record post-treatment pain relief, and provide timely feedback. Physical therapy methods for cancer pain management include microwave, infrared ray, electrical stimulation, heat treatment, etc., which have shown effectiveness in alleviating symptoms of cancer pain. Appropriate physiotherapy methods should be selected based on the location, severity, and other characteristics of the patient’s cancer pain, to achieve specific goals (78). Furthermore, traditional Chinese medicine therapy, guided imagery, music therapy and acupuncture therapy also provide relief in managing malignant pain amongst patients with gynecological malignant tumors.

### 4.3 Mental nursing

Patients with advanced gynecological malignancies often experience negative emotions such as pessimism, anxiety, fear, and depression, which can significantly reduce their quality of life and hinder the effectiveness of treatment (79–81). In order to address the psychological needs of these patients, it is recommended that positive psychological nursing interventions be implemented. These interventions may include establishing a supportive treatment environment, providing education on positive psychology and health, offering empathetic listening and psychological counseling, utilizing relaxation therapy, providing cognitive guidance, facilitating regular experience sharing, and engaging the support of family members (82). Research suggests that creating a comfortable treatment environment can promote emotional well-being, improve treatment outcomes, and enhance patients’ ability to manage cancer-related pain (83). Regular psychoeducation, health education, and cognitive-behavioral intervention are crucial for introducing patients to knowledge about their illness, alleviating anxiety and fear, enhancing treatment confidence, and improving treatment adherence (paragraph 1 modified). This process also enables the examination of the unique psychological characteristics of patients so that targeted interventions can be applied, providing guidance, inspiration, and social support to improve the treatment efficacy of pain in cancer patients. Employing active listening techniques, providing counseling, and promoting regular exchange of experiences can effectively mitigate the emotional burden experienced by patients, reduce their sense of fear and loneliness, facilitate access to social support, bolster confidence in treatment, establish a sense of hope, and increase treatment efficacy. However, the cooperation and understanding of family members are paramount to patients’ treatment and rehabilitation. Establishing an open communication channel and involving family members in the treatment can contribute to their improved understanding of patients’ condition, treatment approaches, and objectives (84). It can also guide them to cooperate with the diagnosis and treatment of the patients, provide more psychological support and care to the patients, thus contributing to improving the treatment effect of the patients’ cancerous pain (83).

### 4.4 Physical nursing

Patients who are at risk of developing pressure sores, respiratory, urinary tract, skin infections, muscle atrophy, and metastasis site symptoms, among others, should receive targeted nursing interventions (85, 86). Those who are confined to bed for prolonged periods or require a fixed position can benefit from an air cushion bed and a turn-over schedule. Regular repositioning should be provided, and skin integrity at compression sites should be monitored to prevent pressure ulcers and infections. Additionally, nursing staff should encourage or assist patients to mobilize their lower limbs effectively to prevent venous thrombosis and reduce the incidence of muscle atrophy (87). Moreover, ensuring optimal oral and perineal hygiene in patients is crucial in decreasing the risk of lung, skin, and pelvic infections. Patients who can move independently should be encouraged to engage in regular mobility by getting out of bed regularly, exposing themselves to fresh air, and moving their limbs appropriately to minimize complications such as infection, thrombosis, and pain-related discomfort (60).

### 4.5 Psychosocial interventions

Psychosocial interventions had meaningful effects on both pain severity and interference (88). Meanhiwle, psychosocial interventions, such as cognitive-behavioral therapy, relaxation and experience intervention, are associated with fewer side effects and should be part of multimodal pain management methods (89). As recommended in a recent Institute of Medicine (IOM) report and guidelines of the American Pain Society, the use of psychosocial interventions as part of a multimodal approach to the treatment of cancer-related pain and the inclusion of experts in psychosocial care should be included as members of the multidisciplinary treatment team (89). In short, psychosocial interventions, including skills instruction and education, can improve cancer pain management.

### 4.6 Continuous care for patients with pain

In recent years, significant advancements have been made in comprehending and treating pain globally. In various regions, extensive research involving continuous care for diverse chronic ailments such as diabetic chronic obstructive pulmonary disease has been conducted and yielded positive outcomes (90). Additionally, studies indicate that patients who underwent continuous care display remarkable enhancement in their ability to manage pain independently at home. Continuous care administration not only elevates the percentage of pain control, but also serves to remind patients to take medication promptly and rectify discrepancies in medication instructions, thereby standardizing the medication guide for patients following discharge from the hospital. Through collaborative interdisciplinary teamwork and pain patient symposia, pain patients are provided with a platform for mutual communication which facilitates their systematic, prompt, and continual treatment, allowing for more accurate self-evaluation of pain score. The above measures can effectively enhance medication compliance in pain patients, prevent and treat adverse reactions more effectively, promote the standardized treatment of pain, increase patient satisfaction, and ultimately improve the relief effect of pain and their quality of life.

## 5 Nursing role in pain management

### 5.1 Standardized pain management

#### 5.1.1 Health care personnel

Pain management is an essential aspect of patient care, yet it often receives inadequate attention in the medical field. Given that pain is widely prevalent among patients, it is crucial to improve healthcare professionals’ knowledge and competence in this area. Various studies have shown that many nurses in level-1 or level-2 hospitals lack sufficient knowledge of pain assessment methods, particularly when managing cancer patients. As a result, effective pain management is often compromised. Moreover, the lack of time and resources, and the wide range of clinical duties, often limit healthcare professionals’ capacity to provide critical health education and resources to pain-suffering patients and their families.

#### 5.1.2 Patient-level factors

The lack of knowledge about pain management among patients and their families often results in patients deliberately enduring pain, as they consider it as a normal experience, without promptly notifying their healthcare providers. This subsequently leads to a delay in receiving the necessary analgesic treatment. Further, several surveys have reported that patients hold a misconception regarding the use of analgesics leading to addiction. This impression causes many patients to discontinue the use of analgesics to avoid dependence and increased drug dosages. The cost of treatment also poses a significant concern for patients, which can result in not adhering to appropriate analgesic therapy.

#### 5.1.3 Lack of medical humanistic thought

As society’s economic status has advanced, the medical profession has similarly progressed. Modern physicians possess exceptional diagnostic and therapeutic capabilities, as well as psychological counseling skills, which enable them to provide personalized treatment regimens tailored to individual patients. It is imperative to emphasize people-oriented thinking and implement standardized pain diagnosis and treatment approaches that prioritize psychological support for patients and their families. This approach aims to assist patients in properly comprehending pain and ultimately achieving effective pain control, thus improving their quality of life. Encouraging patients and caregivers to actively participate in the decision-making process is also recommended as a fundamental component of effective pain management.

### 5.2 Establishment of standardized diagnosis and treatment system in tumor pain clinic

Outpatient pain management is a crucial component in the standardized management of cancer pain diagnosis and treatment, which has yielded some favorable outcomes. Nonetheless, practical observations indicate that some patients continue to experience moderate to severe pain even after being discharged, and the primary reasons for this are as follows:

① Assessing pain in patients in real-time presents a notable challenge. Studies have demonstrated that pain is a subjective phenomenon and that the most reliable and efficacious way to assess it is through the patient’s self-report. Notwithstanding, it is often the case that logistical barriers prevent patients from seeking in-person medical attention, particularly in instances of protracted illness or advanced age. Under such circumstances, family members may be designated as proxies, attending pain clinics in lieu of the patient. Nevertheless, the accuracy of pain assessment and the efficacy of any resultant therapeutic interventions may be compromised by the inability of family members to provide a faithful account of the patient’s pain experience.
② The follow-up work was not satisfactory. At present, the follow-up work is only completed by the hospital alone, while most patients return to the local community or family after discharge from the hospital where there is no mature continuous service. It is difficult to guarantee the follow-up rate at 100%, resulting in a majority of patients still suffer pain at home. It is of great importance to establish medical association between community and tertiary hospitals. Additionally, there are problems that the researchers do not have enough awareness and attention of the continuous care, content of continuous nursing remains simple, health education is lack of individualization, research on continuous nursing is not advisable and the system is not perfect, which are issues in need of attention in the future.
③ Currently, there is a lack of established demonstration wards for standardized pain treatment, resulting in non-uniformity in clinical pain treatment practices and unresolved issues. Despite the emphasis on individualized treatment, the current state of pain treatment is plagued by several challenges, including primary-stage pain treatment in economically less-developed regions, common instances of inadequate pain treatment, and insufficient familiarity and practical experience with analgesics among clinical medical staff. Moreover, the absence of evidence-based medical evidence exacerbates the aforementioned challenges, rendering the standardization of pain treatment elusive. These challenges signal the imperative need for demonstration wards with standardized pain treatment protocols to facilitate effective clinical management, thereby promoting standardization, and mitigating pain occurrences.
④ Continuous care for patients with pain is not performed. In the standardized treatment of pain, nursing staffs play a very important role, for the reason that most cancerous pain is a chronic disease with a long span, and patients often experience aggravation or recurrence of pain due to lack of standard guidance after discharge from hospital. Reports have shown that standardized management of pain-suffered patients should extend beyond the hospital to better ease their suffering. In the future, nursing staffs are supposed to strengthen the continuous care for patients with pain to ensure that the treatment of pain can be effectively continued.

## 5 Conclusion

Gynecological malignancy is a prevalent form of cancer that affects the human body. The development and emergence of this disease is primarily linked to several factors, including reproductive history, sexual age, sexual behavior, menstrual history, endocrine function impairments, infections, smoking, excessive alcohol consumption, and other unhealthy habits (91). The medical field is continuously introducing innovative concepts, methodologies, and technologies, which in turn improves understanding of gynecological malignancies. It is rational to surmise that the disease’s prevention could be achievable in the future (92).

Patients diagnosed with advanced gynecological malignancies, including cervical cancer, endometrial cancer, and ovarian cancer, experience varying degrees of physical pain. This pain significantly impacts the psychological and physiological wellbeing of patients, ultimately compromising the efficacy of treatment. Consequently, the appropriate diagnosis and management of pain are of utmost importance to improve patient outcomes and reduce suffering (93). The management of cancer-related pain involves the implementation of targeted treatments that include drug and non-pharmacological interventions, as dictated by the underlying etiology of the primary disease. Optimal patient care also requires the provision of positive nursing measures to enhance patients’ psychological well-being and foster their confidence in managing pain associated with cancer (94). Implementing drug analgesia and minimally invasive interventional treatment individually can reduce infection, thrombosis and related pain caused by improper posture and hygiene habits. Nurses play a very important role in the pain management of patients with advanced gynecological malignant tumors, including assessing the degree of malignant pain, implementing pain relief measures, assisting clinicians, guiding patients and their families (95). It is imperative to recognize the crucial role of nursing interventions in the management of cancer-related pain in patients with advanced gynecological malignancies. Therefore, nursing staff must possess a profound understanding of advanced gynecological malignancies, including comprehensively assessing the patient and being familiar with pertinent evaluation methods. Additionally, nurses should possess expert knowledge regarding analgesic techniques and strategies, specifically related to the management of pain in the context of advanced gynecological malignant tumor. Nursing interventions should also emphasize the importance of effective communication, and psychological counseling to ensure an evidence-based approach to promote optimal patient outcomes. As a result, nursing staff should adapt to emerging medical models and strive to enhance their overall medical expertise. To improve the quality of life for patients with advanced gynecological malignant tumor, nursing staffs are required to accurately evaluate and record cancer pain incidence, selecting personalized treatment plan, engaging in psychological nursing, advocating health education and effective communication, as well as carrying out appropriate body nursing, all of which can contribute to reducing pain severity and enhancing pain tolerance in patients.

## Author contributions

Original draft preparation, allocation: WW and XH, Manuscript revision, supplement and edition: SL, MJ, and YN. All authors contributed to the article and approved the submitted version.
